# Identification of Metabolites with Antibacterial Activities by Analyzing the FTIR Spectra of Microalgae

**DOI:** 10.3390/life12091395

**Published:** 2022-09-07

**Authors:** Stanislav Sukhikh, Alexander Prosekov, Svetlana Ivanova, Pavel Maslennikov, Anna Andreeva, Ekaterina Budenkova, Egor Kashirskikh, Anna Tcibulnikova, Evgeniia Zemliakova, Ilia Samusev, Olga Babich

**Affiliations:** 1 SEC “Applied Biotechnologies”, Immanuel Kant Baltic Federal University, 236016 Kaliningrad, Russia; 2 Laboratory of Biocatalysis, Kemerovo State University, 650043 Kemerovo, Russia; 3 Natural Nutraceutical Biotesting Laboratory, Kemerovo State University, 650043 Kemerovo, Russia; 4 Department of General Mathematics and Informatics, Kemerovo State University, 650043 Kemerovo, Russia; 5 Higher School of Life Sciences, Immanuel Kant Baltic Federal University, 236016 Kaliningrad, Russia; 6 SEC “Fundamental and Applied Photonics”, Immanuel Kant Baltic Federal University, 236016 Kaliningrad, Russia; 7 Chemistry Department, Kaliningrad State Technical University, 236022 Kaliningrad, Russia

**Keywords:** *Chlorella vulgaris*, *Arthrospira platensis*, FTIR spectra, antimicrobial activity, functional products, biologically active substances, biofuels

## Abstract

Biologically active substances from microalgae can exhibit antioxidant, immunostimulating, antibacterial, antiviral, antitumor, antihypertensive, regenerative, and neuroprotective effects. Lipid complexes of microalgae *Chlorella vulgaris* and *Arthrospira platensis* exhibit antibacterial activity and inhibit the growth of the Gram-positive strain *Bacillus subtilis*; the maximum zone of inhibition is 0.7 ± 0.03 cm at all concentrations. The carbohydrate-containing complex of *C. vulgaris* exhibits antibacterial activity, inhibits the growth of the Gram-positive strain *B. subtilis*, *Bacillus pumilus*; the maximum zone of inhibition is 3.5 ± 0.17 cm at all concentrations considered. The carbohydrate complex of *A. platensis* has antimicrobial activity against the Gram-negative strain of *Escherichia coli* at all concentrations, and the zone of inhibition is 2.0–3.0 cm. The presence of mythelenic, carbonyl groups, ester bonds between fatty acids and glycerol in lipid molecules, the stretching vibration of the phosphate group PO_2_, neutral lipids, glyco- and phospholipids, and unsaturated fatty acids, such as γ-linolenic, was revealed using FTIR spectra. Spectral peaks characteristic of saccharides were found, and there were cellulose and starch absorption bands, pyranose rings, and phenolic compounds. Both algae in this study had phenolic and alcohol components, which had high antibacterial activity. Microalgae can be used as biologically active food additives and/or as an alternative to antibiotic feed in animal husbandry due to their antibacterial properties.

## 1. Introduction

Microalgae cultivation has recently piqued the interest of researchers due to their ability to synthesize a variety of biologically active substances, rapid biomass growth, and the ability to adjust their biochemical composition depending on cultivation conditions. Unlike heterotrophic microorganisms, which need various organic compounds for growth, unicellular photosynthetic organisms produce biomass from fully oxidized inorganic substances and mineral elements due to light energy converted during cultivation photosynthesis. Furthermore, microalgae biomass production technologies do not pollute the environment, use carbon dioxide while releasing oxygen, consume a relatively small amount of water, and can be produced using land resources unsuitable for crop cultivation [1,2,3].

There are currently two main applications for microalgae: biomass production as a biologically active additive (BAA) and cultivation for the subsequent isolation of biologically active substances (BAS) from the biomass [4].

Microalgae are rich in nutrients and biologically active substances, such as proteins, carbohydrates, lipids, polyunsaturated fatty acids, vitamins, pigments, phycobiliproteins, enzymes, etc. Biologically active substances from microalgae can exhibit antioxidant, immunostimulating, antibacterial, antiviral, antitumor, antihypertensive, regenerative, and neuroprotective effects. These compounds are in high demand in medicine, cosmetics, the food industry, fish farming, energy, agriculture, feed, and functional food production [5,6,7,8].

Only a few microalgae species (*Arthrospira (Spirulina) platensis*, *Chlorella* or *Chlorella vulgaris*, *Dunaliella*, *Aphanizomenon*, and *Nostoc*) are currently approved for human consumption. These microalgae are a promising object for large-scale cultivation due to the high content of biologically active substances and the relatively cheap production process. Other microalgae species, such as *Chlamydomonas* sp., *Chlorococcum* sp., *Scenedescmus* sp., *Tetraselmis chuii*, and *Nanochloropsis* sp., have demonstrated their value as ingredients in feed, fertilizers, cosmetics, and aquaculture but do not yet have GRAS status [9,10,11,12,13].

Finding new, unstudied strains of microalgae can broaden the scope of their industrial application and create new opportunities for use. Because of the wide variety of microalgae, their high metabolic flexibility, and the variety of cultivation conditions, their true potential has yet to be fully evaluated. Innovative developments in microalgae production optimization will make their use economically viable and in demand in the future. 

Marine microalgae are microalgae that are used in many parts of the world as food, feed, and fertilizer, as well as a potential renewable resource in medicine and commercial activities. The biostimulatory properties of marine microalgae are being investigated for potential applications in the development of new antibiotics. Many metabolites isolated from marine microalgae have bioactive properties [14]. Bioactive natural products are widely distributed in the plant world, and extracts of various plants, as well as red, green, and brown macro- and microalgae, can be used as natural products [15]. Marine algae are a never-ending source of raw materials for pharmaceuticals, medicine, food processing, and cosmetics [16]. The need for compounds with biological activity for potential pharmaceutical applications or other potentially significant economic properties has resulted in a sharp increase in research into the chemistry of marine microalgae in recent years [17]. Marine microalgae are an important source of bioactive natural substances [18]. Particular attention was paid to the antibacterial activity of marine microalgae against several pathogens [19]. It was demonstrated that various marine microalgae extracts and compounds have antibacterial activity against both Gram-positive and Gram-negative bacteria [19,20]. Antimicrobial compounds derived from marine microalgae are composed of a diverse group of chemical compounds [21,22]. 

The microalgae *Chlorella vulgaris* and *Arthrospira platensis* are of particular interest because they have high potential for use and grow rapidly (doubling time up to 6 h), are more productive at small scales than plants, do not require agriculturally significant land (do not compete with the food industry), use very basic mineral components for growth, use salty sea water, and can grow on wastewater while treating it, use only solar energy, absorb carbon dioxide when growing, contain a large amount of proteins, fats, and carbohydrates [22].

This study sought to identify the antimicrobial metabolites present in microalgae by analyzing their FTIR spectra. The novelty of this study lies in the fact that the antimicrobial activity of biologically active chemical groups identified by IR spectroscopy and found in the proteins, lipids, and carbohydrates of microalgae *Chlorella vulgaris* and *Arthrospira platensis* isolated from the Baltic Sea was investigated for the first time.

## 2. Materials and Methods

### 2.1. Materials

Materials: Zarukka and Tamiya mediums for cultivating medium for cultivating; analytically pure reagents; NaCl (Areolab, Moscow, Russia), chloroform (Areolab, Moscow, Russia), methanol formazin (Areolab, Moscow, Russia), hydrazine sulfate (Areolab, Moscow, Russia), urotropine (Areolab, Moscow, Russia), zinc selenide (Areolab, Moscow, Russia).

### 2.2. Microalgae Samples

Microalgae were collected in the Baltic Sea, Kaliningrad, Russia, in June 2019 as follows.

Microalgae were sampled with a box-shaped bottom sampler developed at the Institute for Biology of Inland Waters of the Russian Academy of Sciences (IBIW) (Borok, Russia), covering a square area of the bottom 160 × 160 mm in size with a maximum immersion depth of 440 mm in bottom sediments; a 400 mm length sample was taken. Immediately after transportation to the shore, test cores were taken using plastic tubes with an inner diameter of 45 mm. The tubes were sealed at both ends and stored in an upright position at +4 °C. In the laboratory, the core was cut lengthwise and halved using two thin stainless-steel plates inserted into the cut. The halves of the core were then divided into transverse samples (slices) with a step of 5–10 mm. All samples were stored at –20 °C in the dark, in plastic bags with air access, from which microalgae samples were taken for research.

Further, pure microalgae cultures were isolated and microalgae strains that can actively accumulate biomass and target products (lipids, proteins, and carbohydrate–mineral complex) and are suitable for cultivation in laboratory conditions were identified.

The collected microalgae were washed to remove impurities and cultivated in 500 mL Erlenmeyer laboratory flasks. The cultivation was carried out on an orbital shaker (Heidolph, Unimax 1010, Schwabach, Germany) at 118 rpm. The algae were dried in a drying oven at T = 40 °C (Memmert, Büchenbach, Germany). Before extraction, the samples were stored at T = 4 °C.

To identify isolates from the enrichment culture strains of microorganisms (microalgae), partial sequences of the 18S and/or 16S rNA encoding gene were determined. DNA from the samples was isolated using DNeasy Plant Pro Kit (Quagen, Limburg, Germany). The primer annealing regions corresponded to forward primer 5′-AACCTGGTTGATCCTGCCAG-3′ and reverse primer 5′-CACCAGACTTGCCCTCCA-3′. The samples were amplified by the qPCRmix-HSreaction mixture (Eurogen, Moscow, Russia) using C1000 Touch system (BioRad, Hercules, CA, USA) and were cloned into pAL2T-vector (Eurogen, Moscow, Russia). After that the recombinant vectors were sequenced by M13 primer system (Eurogen, Moscow, Russia) using 3500 Genetic Analyzer (Applied Biosystems, Waltham, MA, USA). The sequences processing was made by software CLC Genomics Workbench (Quagen, Germany). The comparative analysis was performed with the known sequences from the Genbank database. The results of a comparative analysis of the 18S and/or 16S rNA gene sequence showed that the following microalgae were isolated from the Baltic Sea: *Chlorella vulgaris* and *Arthrospira platensis* [23,24].

### 2.3. Extraction of Protein Concentrate, Lipid and Carbohydrate Complexes

To extract the protein complex, 2 g of dried algae were dissolved in 40 mL of distilled water and incubated at 4 °C for 16 h. The solution was centrifuged at 9000 rpm for 20 min at 4 °C (Thermo Scientific™ Heraeus™ Megafuge™ 16 Centrifuge Series, Waltham, MA, USA. After centrifugation, the obtained precipitate was treated with acid (HCl) and alkali (NaOH) at concentrations of 0.3 M. A solid to solvent ratio of 1:15 was used and the solutions were stirred for 1 h at 4 °C and then centrifuged at 9000 rpm for 20 min at 4 °C. The resulting precipitate was dried at 40 °C for 18 h, and its protein content was analyzed. The protein content was also measured in the supernatant, the values were summarized.

The Folch method was used to extract the lipid–pigment complex. For this, 2 mL of a chloroform:methanol (2:1 by volume) mixture was used per 100 mg of dry biomass. Next, the sample was sonicated for 30 min to extract the lipid fraction. After sonication, 0.25 volumes of 0.9% sodium chloride solution were added to the sample, and the mixture was intensively stirred. After phase separation, the organic phase was separated and evaporated using a rotary evaporator to constant weight. The dry weight of the lipid fraction was determined by weighing. The lipid content *W*_l_ (%) was determined using the following formula:

$$W_{l}=\frac{m_{l}}{m_{b}}\times 100\;\%$$

where *m*_l_—mass of extracted lipids; *m*_b_—mass of dry biomass.

Acid hydrolysis was used to extract the carbohydrate–mineral complex. Sulfuric acid with a sulfuric acid concentration of 5.0% was used. Hydrolysis was performed at a temperature of 121 °C for 20 min at a pressure of 1 atm. The loading volume of dried microalgae biomass during the experiment was 50 g/L. After the hydrolysis process, the samples were cooled at room temperature and centrifuged at 7000 rpm for 5 min. The total concentration of carbohydrates was analyzed using the phenol–sulfuric acid method. A reagent was prepared for analysis using the phenol–sulfate method. To prepare it, 5 g of phenol was added to 100 mL of distilled water. The standard curve was plotted with different concentrations of D-glucose. In total, 50 µL of the reagent was added to 50 µL of the sample and followed by 2 mL of concentrated sulfuric acid. The solution was kept at room temperature until an orange color was obtained. The presence of the carbohydrate complex was determined spectrophotometrically at a wavelength of 490 nm.

### 2.4. Determination of the Antibiotic Activity of Microalgae 

The antibiotic activity was determined by the disk diffusion test [19]. A bacterial suspension was applied to the surface of the nutrient medium with agar in Petri dishes within 15 min after preparation. Petri dishes were innoculated manually by applying the suspension evenly with streaking movements on the entire surface of the agar in three directions so that there were no gaps between the strokes. Six-millimeter disks with the antibiotic ampicillin (control), lipid, carbohydrate and protein complexes (20 mL) were applied to the agar surface within 15 min after inoculation of the bacterial suspension. The contact of the disks with agar was complete and tight. The disks were not moved after being applied to the agar surface because the antibiotic and the studied complexes isolated from microalgae diffused into the medium very quickly. The dishes were incubated for 24 h at 37 °C, and the inhibition zone was measured in centimeters (cm). 

Microalgae lipid solution for determination of antimicrobial activity was prepared in 5% Tween 20 (polysorbate-20) emulsifier solution by adding 32 mg of lipids to 1 mL of 5% Tween 20. Polysorbates are very strong emulsifiers. They weaken the surface active tension between water and oil, causing the process of solubilization. Solubilization means the dissolution of lipids in water and facilitates the diffusion of lipids into agar media, exhibiting antibacterial properties [25].

*A. platensis* and *C. vulgaris* lipid complex samples, that included all isolated lipids, were tested at 50, 75, and 100 mg/mL. Discs with ampicillin (10 mg/mL) were used as controls to assess the inhibition zones. The tests performed in triplicate (biological triplicates). Two different Gram-positive bacteria (*Bacillus subtilis* (B-7918) and *Bacillus pumilus* (B-1133) and one Gram-negative strain of *Escherichia coli* showed insignificant differences in the diameter of the zone of inhibition when interacting with the extract isolated from microalgae. *B. subtilis* is only a pathogen in severely immunocompromised patients as a result of severe illness [26]. However, the study of the antimicrobial activity of microalgae in relation to this strain is of clinical interest. In this regard, the effect of microalgae on this Gram-positive bacterium was studied. 

The isolated and purified extracts were diluted from 100 mg/mL to 1 mg/mL. Three concentrations of organic complexes (100, 10, 1 mg/mL) were formed and tested on pathogenic and opportunistic microorganisms. To completely remove the effect of solvents on microorganism growth, preliminary measures were taken to remove them from the composition of the tested organic complexes. A rotary evaporator was used to remove n-hexane residues from the lipid complex. 

### 2.5. FTIR Spectroscopy

FTIR spectroscopy of solid microalgae samples: A dry microalgae sample was mixed with KBr (Pike Technologies, Madison, WI, USA) and ground in an agate mortar to obtain a fine fraction. The resulting mixture was compressed into a transparent tablet. The FTIR spectra of the obtained tablet were measured on an IRPrestige-21 IR spectrometer (Shimadzu, Kyoto, Japan) in the range of 500–4000 cm^−1^.

FTIR spectroscopy of liquid microalgae samples: In total, 0.11 g of KBr powder (Pike Technologies) was impregnated with 0.5 mL of microalgae sample solution. Next, the powder with the sample was dried in an oven (Memmert, Schwabach, Germany) at a temperature of 50 °C until complete evaporation of the liquid within 40 min. The resulting dry powder was triturated in an agate mortar to grind the fraction. The resulting mixture was compressed into a transparent tablet. The tablet FTIR spectra were measured on an IRPrestige-21 IR spectrometer (Shimadzu, Kyoto, Japan) in the range of 500–4000 cm^−1^ [27].

### 2.6. Statistical Analysis

The data were subjected to analysis of variance (ANOVA) using Statistica 10.0 (StatSoft Inc., 2007, Tesla, WV, USA). Post hoc analysis (Duncan’s test) was undertaken to identify samples that were significantly different from each other. The equality of the variances of the extracted samples was checked using the Levene test. Differences between means were considered significant when the confidence interval is smaller than 5% (*p* < 0.05).

## 3. Results

Table 1, Table 2, Table 3 and Table 4 present the antimicrobial activities of the *C. vulgaris* and *A. platensis* lipid and carbohydrate complexes.

**Figure 1:**
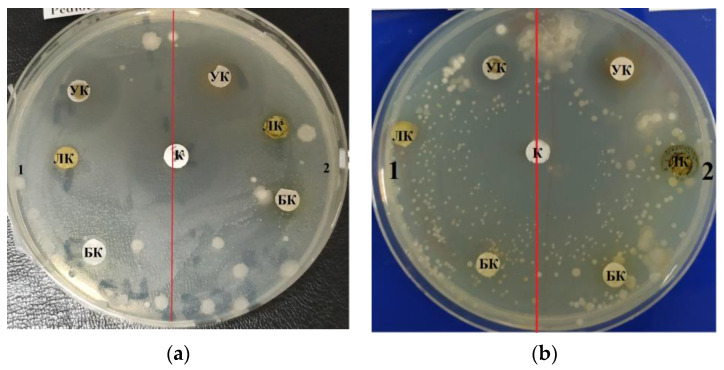
Zones of inhibition on a monolayer of (**a**) *B. subtilis* and (**b**) *E. coli* cells: 1—*A. platensis*. 2—*C. vulgaris*. K—Control. БK—Protein complex. ЛK—Lipid complex. УK—Carbohydrate complex.

**Figure 2:**
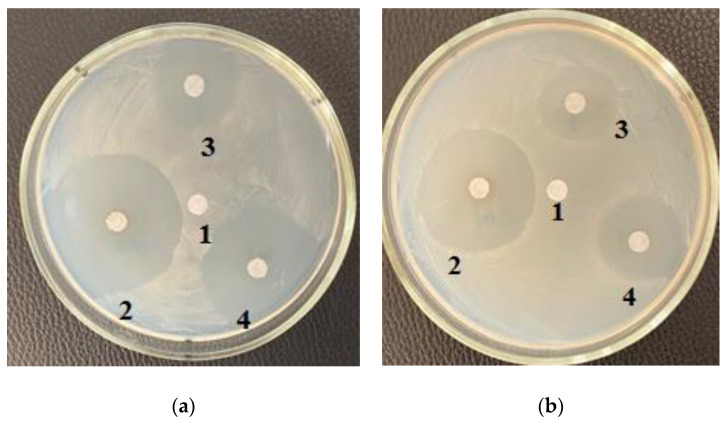
Effect of the purified (**a**) carbohydrate and (**b**) lipid complexes isolated from *A. platensis* on the *B. subtilis:* 1—Control (without complex); 2—concentration 100.0 mg/mL; 3—concentration 10.0 mg/mL; 4—concentration 0.1 mg/mL.

**Figure 3:**
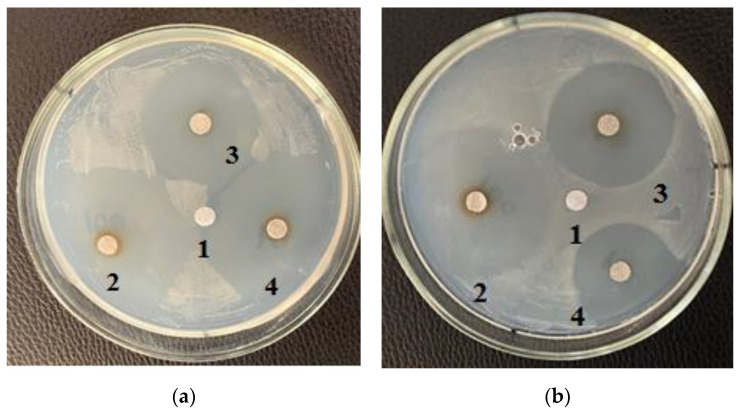
Effect of the purified (**a**) carbohydrate and (**b**) lipid complexes isolated from *C. vulgaris* on the *B. subtilis:* 1—Control (without complex); 2—concentration 100.0 mg/mL; 3—concentration 10.0 mg/mL; 4—concentration 0.1 mg/mL.

Figure 1, Figure 2 and Figure 3 depict the zones of inhibition of *B. subtilis* and *E. coli* by *C. vulgaris* and *A. platensis* lipid and carbohydrate complexes.

**Figure 4:**
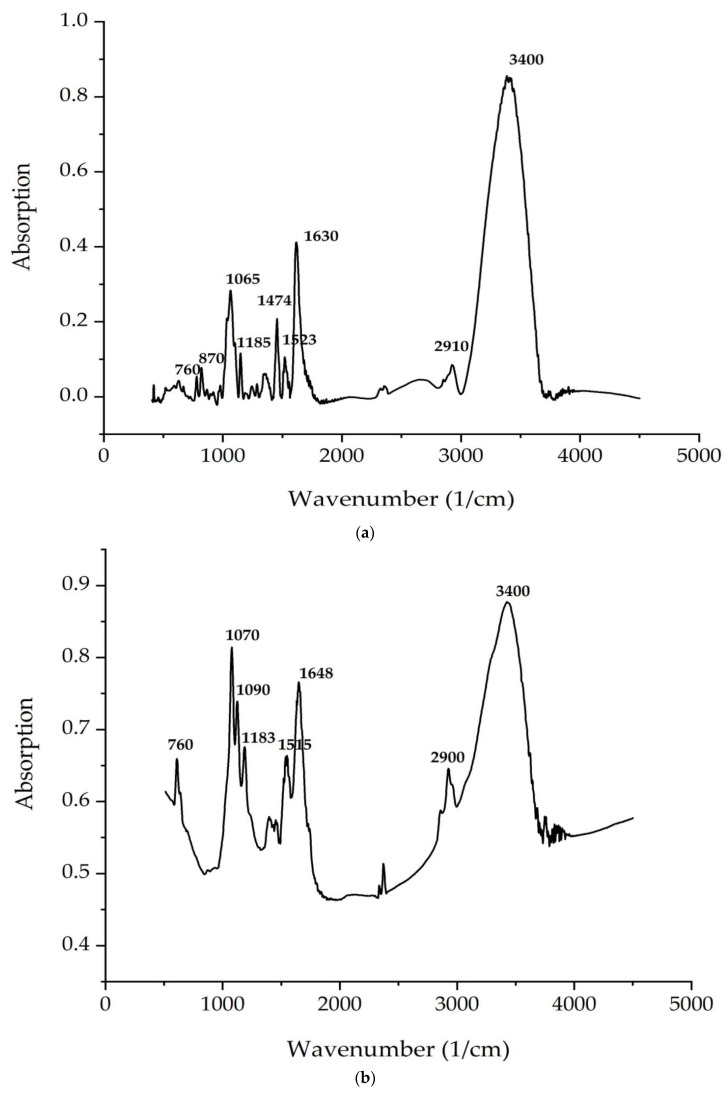
FTIR spectra of the (**a**) *C. vulgaris* and (**b**) *A. platensis* lipid complex.

**Figure 5:**
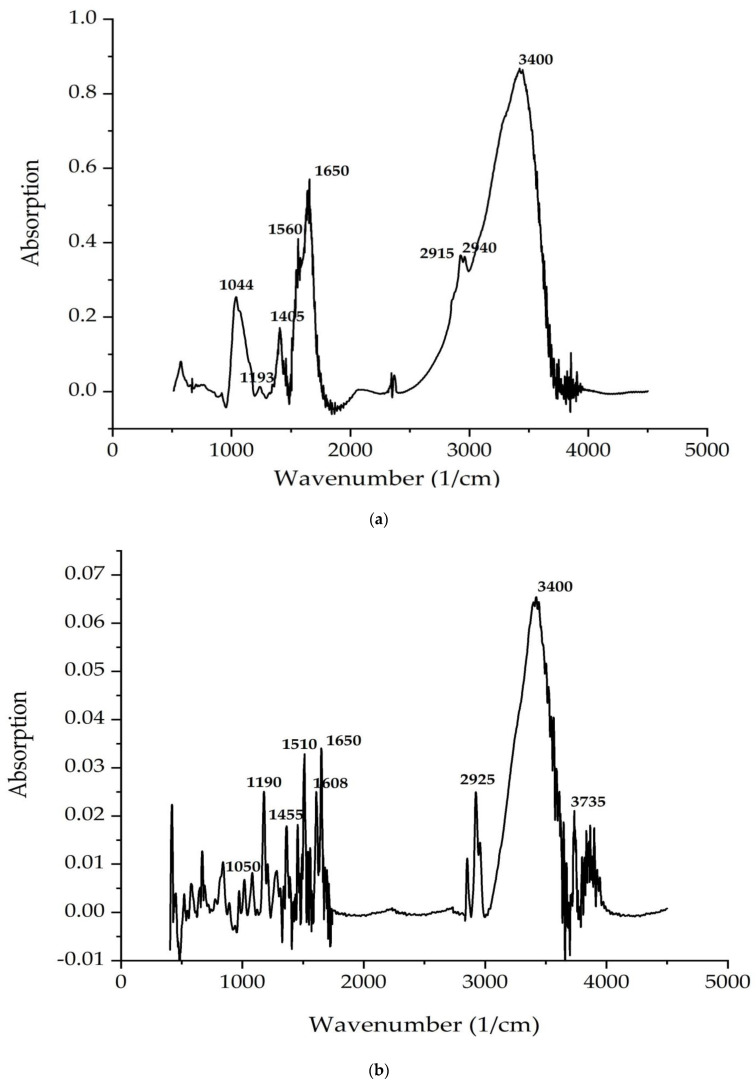
FTIR spectra of the (**a**) *C. vulgaris* and (**b**) *A. platensis* carbohydrate complex.

Figure 4 and Figure 5 demonstrate FTIR spectra of the *C. vulgaris* and *A. platensis* lipid and carbohydrate complexes. The most intense characteristic oscillation frequencies are indicated in the spectra of Figure 4 and Figure 5.

Table 5 and Table 6 shows the theoretical interpretation of the IR spectra of the *C. vulgaris* and *A. platensis* lipid and carbohydrate complexes (Figure 4 and Figure 5).

## 4. Discussion

Moderate antimicrobial activity was detected when three concentrations of organic complexes that were isolated and purified from microalgae were analyzed. Taking into account the revealed activity only in the carbohydrate and lipid complexes, tests were carried out with various concentrations of these substances.

Sea microalgae were selected based on morphological features characteristic of the edible microalgae *A. platensis* and *C. vulgaris*, which were additionally identified using DNA sequences. Only edible microalgae were studied because, in accordance with the proposed hypothesis, the authors intended to obtain a feed additive based on these microalgae for livestock and poultry if the antibacterial activity of the lipid and carbohydrate complexes is confirmed. It is not possible to feed animals with inedible microalgae species.

Lipids play an important role in the metabolism and growth of microalgae, acting as a reservoir of energy and carbon [28,29]. Microalgae contain both polar and neutral lipids. Polar lipids such as phospholipids and glycolipids generated by chloroplasts dominate the cell wall and organellar membranes, while neutral lipids (mono-, di-, and triacylglycerol) are stored in cell organelles [29,30]. The lipid profile of microalgae is wide and ranges from 2 to 77% depending on the species and environment. In *C. vulgaris*, it ranges from 5 to 58% of dry matter. *C.* vulgaris produces more lipids (60–68%) when cultivated under mixotrophic conditions [29,31].

*Arthrospira platensis* was grown on Zarrouk medium, and *Chlorella vulgaris* was grown on Tamiya medium. The biomass yield of each microalga after 7–14 days of cultivation was 30–40 million cells/mL. Protein extraction from *Arthrospira platensis* biomass was 60.03 ± 1.80%; from *Chlorella vulgaris* biomass, 56.20 ± 1.68%; the extraction of lipids from the biomass of *Arthrospira platensis* was 7.23 ± 0.21%; from the biomass of *Chlorella vulgaris*, 16.24 ± 0.48%; the extraction amounts of carbohydrates from microalgae biomass were 11.44 ± 0.34% and 11.22 ± 0.83% for *Arthrospira platensis* and *Chlorella vulgaris*, respectively.

The purified lipid complex isolated from *C. vulgaris* demonstrated antimicrobial activity against a Gram-negative strain of the bacterium *E. coli*, as evidenced by the zones of inhibition results shown in Table 1. The maximum effective inhibitory concentration of the lipid complex samples against *E. coli* was observed at a concentration of 10.0–1.0 mg/mL; the area of the zone was 1.3 ± 0.06 cm^2^. At a higher concentration (100.0 mg/mL), inhibition was less pronounced and amounted to 1.0 ± 0.05 cm (Figure 1a). *C. vulgaris* lipid complex samples also inhibited the growth of Gram-positive *B. subtilis* and *B. pumilus* strains; the maximum zone of inhibition was 2.2 ± 0.11 cm at a concentration of 100.0 mg/mL (Table 1). Table 2 shows the results of measuring zones of inhibition at different concentrations of the purified lipid complex obtained from *A. platensis* samples. The lipid complex was found to be effective against E. coli across the entire concentration range examined, 100.0–10.0–1.0 mg/mL. The lipid complex of microalgae samples also suppressed the growth of the Gram-positive strain B. subtilis; the maximum zone of inhibition was 0.7 ± 0.03 cm at all concentrations used. No increase in the inhibition zone area was found with increasing concentration of the extract (Figure 1b).

Carbohydrates such as starch, glucose, sugar, and polysaccharides are commonly used as energy and carbon storage in microalgae. The most common polysaccharides of *C. vulgaris* are starch, composed of amylose and amylopectin, followed by polysaccharide cellulose in the cell wall. The total carbohydrate content of *C. vulgaris* can reach 12–55% of dry weight when grown under unfavorable conditions, especially those with limited nitrogen [29,32]. *Arthrospira platensis* contains proteins (60%), carbohydrates (15%), lipids, phycobiliproteins, carotenoids, vitamins, and minerals [33].

Table 3 shows the zones of inhibition results of various concentrations of purified carbohydrate complex derived from *C. vulgaris*. Samples of the purified carbohydrate complex were effective against *E. coli* at concentrations of 100.0–10.0–1.0 mg/mL. Samples of the purified carbohydrate complex of *C. vulgaris* exhibited antibacterial activity against the Gram-positive *B. subtilis* strain; the maximum zone of inhibition was 3.5 ± 0.17 cm at a concentration of 1.0 mg/mL (Figure 3a). A similar effect was also observed against *B. pumilus*; the maximum zone of inhibition was 3.5 ± 0.17 cm at a concentration of 100.0 mg/mL. Table 4 shows the zones of inhibition results of various concentrations of purified carbohydrate complex obtained from *A. platensis*. Antimicrobial activity against *E. coli*, *B. pumilus*, and *B. subtilis* was revealed. The greatest inhibitory effect of samples of the carbohydrate complex of microalgae was observed at a concentration of 100.0 mg/mL, and the inhibition zone was 3.0–4.2 cm.

Other studies have also shown the antimicrobial activity of various extracts from *C. vulgaris* biomass [34,35]. In this study [34], an aqueous extract of C. vulgaris showed antibacterial activity against both Gram-negative (*E. coli*) and Gram-positive (*S. aureus*) bacteria. It was found that an aqueous extract at a concentration of 150 mg/mL exhibited antimicrobial activity against E. coli, and the diameter of the inhibition zone was 2.4 cm. The highest antimicrobial activity against E. coli had a protein fraction obtained by TCA from an aqueous extract, with MICs in the range of 32.5–65 mg/mL [34]. In other studies, extracts of green unicellular algae showed pronounced antagonistic activity against numerous opportunistic and pathogenic bacteria [33,34,35,36,37].

It has been established that *Chlorella vulgaris* synthesizes silver nanoparticles in the dark, for which antimicrobial activity was studied on three pathogenic microorganisms: Gram-negative bacterium *Erwinia*, Gram-positive bacterium *Bacillus* sp. and pathogenic fungus *Candida*. The specific antibiotics penicillin (10 mg), tetracycline (30 mg), and streptomycin (10 mg) were used as controls. The synthesized AgNP solutions had an inhibitory effect on all tested microorganisms, with the following order of *Bacillus*, *Erwinia*, *Candida* in terms of increasing the radius of the zone of inhibition. The effect of nanoparticles on all test organisms was more pronounced than the effect of silver nitrate and penicillin. This effect can be explained by the fact that silver nanoparticles penetrated the bacterial cell wall and damaged them due to their interactions with compounds containing phosphorus and sulfur, including DNA [38].

Previous studies have reported that chlorellin (a mixture of fatty acids) from *C. vulgaris* exhibited inhibitory activity against both Gram-positive and Gram-negative bacteria [29,39]. Linolenic acid in ethanolic extracts of *C. vulgaris* also showed antibacterial activity against *Staphylococcus aureus* (a common cause of skin infections) and *Salmonella typhi* (causative agent of typhoid fever). This property suggests there is a possibility to use *C. vulgaris* as a natural antibiotic and that that it could be a promising alternative to traditional synthetic drugs with a wider spectrum of action against pathogenic infections [29,40]. One of the most important polysaccharides found in *C. vulgaris* samples, β-1,3-glucan, has recently gained popularity among researchers due to its dietary qualities and therapeutic properties on human health, such as scavenging free radicals and lowering blood lipids [29,41]. *C. vulgaris* polysaccharides have also been found to exhibit other health benefits, such as antitumor, antiviral, and strong immunomodulatory effects, indicating potential medical applications [42].

In this regard, our results confirm and reveal the potential of *C. vulgaris* extracts for the production of natural fungicides and bactericides. The use of *C. vulgaris* extracts with antimicrobial properties as an alternative to antibiotics in animal husbandry reduces the risk of emergence of antibiotic-resistant bacteria and transmission of antibiotic-resistant pathogens to humans [43,44,45]. Moreover, *C. vulgaris* is a source of valuable biomass suitable for use in the production of various bioproducts [46].

It is also known that *A. platensis* has the potential to act as an antimicrobial agent. The antimicrobial activity of *Arthrospira platensis* against four types of Gram-positive (*Micrococcus luteus*, *Staphylococcus aureus*, *Bacillus cereus*, *Listeria monocytogene* sp.) and two Gram-negative (*Salmonella typhi*, *Pseudomonas aeruginosa*) bacteria was shown by determining zones of inhibition. *A. platensis* showed antibacterial potential against all the studied microorganisms. The inhibition zones of the tested strains varied from 0.9 to 1.3 cm [33].

Extracts of *A. platensis* can be effectively used in aquaculture to combat bacterial diseases [47]. Cyanobacteria (blue-green algae) have unique biochemical properties and are a potential source of biologically active secondary metabolites. Cyanobacteria produce intracellular and extracellular metabolites with antialgae, antibacterial, antifungal, and antiviral activity [33,47]. Spirulina is an ideal bioresource due to its richness in protein, phycocyanin, essential amino acids, polysaccharides, carotenoids, minerals, vitamins, and essential fatty acids [33,47]. It is also rich in vitamins, minerals, carbohydrates, and gamma-linolenic acid. Spirulina is of interest not only because of its nutritional value, but also because it has the potential to be used in the development of pharmaceutical preparations. Spirulina has therapeutic effects as a growth promoter, probiotic, and immune system enhancer in animals, including fish [47]. Phycocyanin is the main biologically active substance in spirulina, and its content ranges from 10 to 15% of dry weight. Spirulina samples exhibit antiviral and antioxidant properties against human pathogens [47].

Thus, algae and cyanobacteria, in addition to their nutritional value, have a wide range of other properties and characteristics, including antimicrobial ones. The study [35] showed that *Ascophyllum nodosum* had the highest inhibitory effect on the growth of E. coli compared to other algal species. The inhibitory effect of *A. nodosum* on the growth of *E. coli* is most likely due to functional algae compounds such as phlorotannins, which are polyphenols with bacteriostatic and bactericidal activity [35,48]. *Lithothamnium calcareum* also showed antimicrobial activity due to the ability of algae to produce metabolites of antimicrobial drugs, such as diterpenes [49], monoterpenes [50], phenolic compounds [51], sterols [52], polysaccharides [53], and fatty acids [52,54]. Studies have confirmed the antimicrobial activity of natural extracts derived from algae and cyanobacteria [55]. *A. nodosum* and *C. vulgaris*, at the highest concentration tested (1:4), have been found to have significant antibacterial activity.

According to Figure 4 and Figure 5, the bands correspond to asymmetric and symmetric stretching vibrations of the methylene groups in the range from 2938 to 2835 cm^–1^. Asymmetric and symmetric bands were designated as Ʋ (CH2) and Ʋ s(CH2), respectively.

At 1680 cm^−1^ (*C. vulgaris*) and 1700 cm^−1^ (*A. platensis*), there is a very important band associated with the stretching vibrations of the carbonyl group (C = O), which is part of the ester bond between fatty acids and glycerol in lipid molecules. However, this type of bond can also be formed by the peroxidation of fatty acid chains. Therefore, it can be assumed that an increase in the intensity of this band indicates an increase in lipid oxidation in the sample. The following bands can be noted: a series of bands from 1474 to 1395 cm^−1^ (*A. platensis*, *C. vulgaris*) (deformation vibrations of the methylene groups of fatty acids), 1183 cm^−1^ (*A. platensis*) and 1185 cm^−1^ (*C. vulgaris*) (stretching vibration of group C–O ester bond of lipids), and 1044 cm^–1^ (*A. platensis*) and 1045 cm^–1^ (*C. vulgaris*) (stretching vibration of the phosphate group PO_2_) [56].

The analysis of the IR spectra of the *C. vulgaris* and *A. platensis* lipid complexes confirms the presence of neutral lipids, glyco- and phospholipids, as well as unsaturated fatty acids, such as γ-linolenic acid, in the composition. As shown in Figure 5a,b, spectrum peaks are typical of saccharides. Absorption in the region of 1200–650 cm^−1^ is attributed to the stretching vibrations of the C–O-group of carbohydrates, a number of authors consider the bands at 1090 and 1044 cm^−1^ to be absorption bands of cellulose, and bands at 2940 cm^−1^ are attributed to starch. The band at 850 cm^−1^ indicates type I glycosidic bonds [57]. Absorption in the region 1040–1200 is associated with the presence of pyranose rings (configurations) [58].

The peaks recorded at 3400 cm^−1^ and 2940 cm^−1^ indicate the stretching vibrations of the hydroxyl groups that make up carbohydrates, and the asymmetric stretching vibrations –CH2– of their molecules, respectively. The identified organic compounds showed good antimicrobial activity. In the present study, both algae contained phenolic and alcohol compounds that were responsible for antibacterial activity [59]. Our results correlate well with data reported in other studies [60,61,62].

The study [60] used Fourier transform infrared spectroscopy (FTIR) to analyze a lipid extract from a natural isolate of *C. vulgaris*. To assess the productivity of the strain, a reference strain was obtained. Lipids were extracted using the Bligh and Dyer method, and the samples were subjected to FTIR analysis to examine the absorption spectra in the range from 2000 to 3000 cm^−1^ to confirm the presence of lipids. For a natural *C. vulgaris* isolate, lipid samples were analyzed using FTIR. As a result, they had eight clear bands in the range of wavenumbers from 4000 to 5000 cm^−1^. These bands were previously identified based on reference standards [61] and published FTIR spectra for specific molecular groups. The results were interpreted. The absorption peak at 3949.70 cm^−1^ and 3840.10 cm^−1^ indicates the presence of primary amines and very weak secondary amines. Peaks at 3383.39 cm^−1^, which indicate OH group absorption, reveal the presence of a strong alcohol group.

The absorption of C–H appearing at 2144.99 cm^−1^ indicated the presence of lipids. Absorption at 2920 cm^−1^ indicated an alkyne group, and that at 1638.05 cm^−1^ and 1523.62 cm^−1^ indicated C=C absorption, which is an alkene group.

In particular, the absorption at 1273.16 cm^−1^ indicated the presence of a strong acid. An absorption at 1149.44 cm^−1^ indicates an ester group; the remaining absorption range at 589.99 cm^−1^, 699.12 cm^−1^, and 1032.75 cm^−1^ indicates the presence of a strong alkyl halide. The presence of peaks at 3383.39 cm^−1^, indicating the absorption of OH groups and indicating the presence of a strong alcohol group, determines the presence of antimicrobial properties of the *C. vulgaris* lipid complex under study.

All *Chlorella* microalgae species are very important for lipid and biomass production, are adaptable to a variety of environmental conditions, are tolerant of high CO_2_ concentrations, and are useful in industrial wastewater treatment and wastewater treatment systems due to the phenolic compounds found in the microalgae lipid fraction exhibiting significant antimicrobial activity [20].

The study [62] showed that the FTIR profile of the *C. vulgaris* lipid fraction revealed various chemical functional groups. The FTIR spectrogram of the lipid complex is dominated by a broad strong absorption band at 3465 cm^−1^ attributed to the hydroxyl group (-OH absorption), whereas the spectral peaks located in the range of 4000–3400 cm^–1^ can be attributed to alcohol and acids [62], which exhibit the highest antimicrobial properties. Strong peaks in the range from 1628 to 1428 cm^−1^ indicate asymmetric and symmetric stretching vibrations, which are related to carboxylate anions (COO-) [62]. These significant spectral peaks may help in elucidating the structure of microalgae lipid complexes for recognizing metal–carboxylate interactions, according to Flórez-Fernández et al. [63]. The spectral band at about 2923–2854 cm^–1^ is consistent with the band obtained by Aprilliza [64] and is associated with aliphatic –CH absorption, as well as symmetric and asymmetric absorption (C–H) CH_2_, in addition to aromatic and/or vinyl C–H absorption and (CH)-anomer absorption [65].

The FTIR spectrogram of the *C. vulgaris* lipid complex also illustrates the peak of C–H stretching vibrations recognizing alkanes, C=O indicates a carbonyl group, and COO- stretching vibrations are attributed to carboxylate, as well as C–O-C stretching vibrations. Peaks reaching the range of 1090–1030 cm^−1^ are related to the C–O absorption of the pyranosyl ring, asymmetric C–O-C absorption (glycosidic bond), and C–C absorption, which are attributed to the structure of the alginate saccharide [66,67]. In addition, absorbances of the C=O group were recorded at 1734 cm^−1^, as reported by Carpenter and Saharan [67]. The present results are consistent with those of Cardenas-Jiron et al. [68] and Bouissil et al. [69]. According to Gomaa et al. [70], the spectral band at 848 cm^−1^ confirms the presence of sulfate groups in the fucoidan of phenolic compounds. Peaks at around 600 cm^−1^ may be associated with symmetric and asymmetric O=S=O deformation, as reported by Flórez-Fernández et al. [63].

Noura El-Ahmady El-Naggar et al. investigated the production and properties of *Chlorella vulgaris* carbohydrates (polysaccharides) [71]. FT-IR analysis of the extracted polysaccharides showed the presence of N–H, O–H, C–H, –CH3,>CH2, COO–1, S=O, and C=O functional groups. Spectral analysis showed the presence of proteins, nucleic acids, and chemical groups (ether, carbonyl, carboxyl, and amine) [72].

The study [72] used the IR spectrum to identify the functional group of active components based on the peak value in the infrared region. The crude powder of *U. lactuca* was passed through an IR spectrometer, and the functional groups of the components were separated based on the ratio of their peaks. The results of the FTIR analysis of microalgae showed different peaks at 620.15 and 848.99, with the functional group being alkyl halides; 928.18—the functional group is carboxylic acid; 1086.14 and 1144.12—the functional group is aliphatic amine; 1384.42—the functional group is nitromethane; 1428.88—the functional group is a group representing aromatic compounds; 1634.33—the functional group is amide; 2295.04—the functional group is nitrile; 3406.60—the functional group is alcohol. *U. lactuca* sample contained phenols. Similarly, crude *G. corticata* powder was passed through the IR spectrum and the functional groups of the components were separated based on the ratio of their peaks. The results of FTIR analysis showed, as in *G. corticata*, different peak values: 3321.46—the functional groups are alcohols and phenols; 2925.49—the functional groups are alkanes; 2084.83—the functional groups are allenes, ketenes, cyanates, and isothiocyanates; 1647.84—the functional groups are isamides; 1471.30—the functional groups are isaromatic compounds. The *G. corticata* sample contained isaliphatic amines with the functional group (1116.62), primary and secondary amines with the functional group (874.41), and alkyl halides with the functional group (750.98, 712.56, 657.10, and 617.26) [72].

## 5. Conclusions

As part of our studies, we established, for the first time, that samples of the lipid and carbohydrate complexes of the Baltic Sea microalgae (*Chlorella vulgaris* and *Arthrospira platensis*) exhibit pronounced antimicrobial activity against three pathogens at once: the Gram-positive bacteria *Bacillus subtilis* and *Bacillus pumilus* and a Gram-negative strain of *Escherichia coli*. The zone of pathogen inhibition under the action of separately isolated lipid and carbohydrate complexes of the studied microalgae was up to 4.2 cm. For comparison, other researchers, as a rule, study the complex effect of microalgae extracts on any one pathogen or on other types of pathogens, and the maximum zone of inhibition is less (2.4 cm). Moreover, in none of the publications we reviewed did we find results confirming the antibacterial activity of separate lipid and carbohydrate complexes through the use of IR spectroscopy, which makes it possible to identify the active groups of these complexes, identify them and prove the effect of certain chemically active groups on pathogenic and opportunistic microorganisms. None of the studies described the antibacterial activity of two biologically active complexes (carbohydrates and lipids) of microalgae isolated from the Baltic Sea at once. New data on the effect of different concentrations of isolated complexes on their antibacterial activity was obtained, and rational concentrations of lipid and carbohydrate complexes, at which they exhibit the greatest activity, was established.

Thus, the marine algae extracts have demonstrated various biopotential activities, such as antibacterial activity [19]. Numerous researchers have demonstrated the antibacterial activity of red, brown, and green algae against both Gram-positive and Gram-negative bacteria [73]. The marine environment contains a source of functional materials, including polyunsaturated fatty acids, polysaccharides, essential minerals, vitamins, antioxidants, enzymes, and bioactive peptides [74]. FTIR is a useful tool for measuring a wide range of chemical components in plants and algae, as well as for revealing some qualitative aspects of organic compounds [72]. This study used FTIR to identify the functional groups of algae. Alcohols are commonly found to have antimicrobial properties against both Gram-positive and Gram-negative bacteria [19]. Phenolic compounds demonstrated good antimicrobial activity [75]. Both algae contained phenolic and alcohol compounds, which were responsible for antibacterial activity. *C. vulgaris* was compared to *A. platensis* in this study, with *C. vulgaris* being more active. Microalgae hold great potential for use as biologically active food additives, with effective forms with a highly antagonistic effect against opportunistic microorganisms in animal husbandry, and for use as a substitute for antibiotics in animal feed.

## Figures and Tables

**Table 1:** Antimicrobial activity (zone of inhibition, cm) of the *C. vulgaris* lipid complex.

Strain	Control (Ampicillin)	Lipid Complex		
		100.0 mg/mL	10.0 mg/mL	1.0 mg/mL
*E. coli*	3.0 ± 0.09	1.0 ± 0.03	1.3 ± 0.04	1.3 ± 0.04
*B. pumilus*	3.0 ± 0.09	2.2 ± 0.06	1.9 ± 0.05	1.9 ± 0.05
*B. subtilis*	3.0 ± 0.09	1.6 ± 0.04	1.5 ± 0.04	1.0 ± 0.03
Mean	3.0 ± 0.09	1.6 ± 0.04	1.5 ± 0.04	1.4 ± 0.03

All values in rows do differ significantly (*p* < 0.05) as assessed by the post hoc test (Tukey test). Data presented as a mean ± SD (*n* = 3).

**Table 2:** Antimicrobial activity (zone of inhibition, cm) of the *A. platensis* lipid complex.

Strain	Control (Ampicillin)	Lipid Complex		
		100.0 mg/mL	10.0 mg/mL	1.0 mg/mL
*E. coli*	3.0 ± 0.09	1.0 ± 0.03	1.0 ± 0.03	1.0 ± 0.03
*B. pumilus*	3.0 ± 0.09	0.7 ± 0.02	0.7 ± 0.02	0.7 ± 0.01
*B. subtilis*	3.0 ± 0.09	0.3 ± 0.01	0.3 ± 0.01	0.3 ± 0.01
Mean	3.0 ± 0.09	0.6 ± 0.01	0.6 ± 0.01	0.6 ± 0.01

All values in rows do differ significantly (*p* < 0.05) as assessed by the post hoc test (Tukey test). Data presented as a mean ± SD (*n* = 3).

**Table 3:** Antimicrobial activity (zone of inhibition, cm) of thse *C. vulgaris* carbohydrate complex.

Strain	Control (Ampicillin)	Carbohydrate Complex		
		100.0 mg/mL	10.0 mg/mL	1.0 mg/mL
*E. coli*	3.0 ± 0.09	3.0 ± 0.09	3.3 ± 0.09	3.3 ± 0.09
*B. pumilus*	3.0 ± 0.09	3.5 ± 0.10	3.2 ± 0.09	3.2 ± 0.09
*B. subtilis*	3.0 ± 0.09	2.2 ± 0.16 *	2.2 ± 0.06 *	3.5 ± 0.10
Mean	3.0 ± 0.09	2.9 ± 0.11	2.9 ± 0.08	3.3 ± 0.10

Values in rows followed by the symbol “*” do differ significantly (*p* < 0.05) as assessed by the post hoc test (Tukey test). Data presented as a mean ± SD (*n* = 3).

**Table 4:** Antimicrobial activity (zone of inhibition, cm) of the *A. platensis* carbohydrate complex.

Strain	Control (Ampicillin)	Carbohydrate Complex		
		100.0 mg/mL	10.0 mg/mL	1.0 mg/mL
*E. coli*	3.0 ± 0.09	3.0 ± 0.09	2.4 ± 0.07	2.0 ± 0.06 *
*B. pumilus*	3.0 ± 0.09	3.7 ± 0.11	2.4 ± 0.10	2.0 ± 0.06 *
*B. subtilis*	3.0 ± 0.09	4.2 ± 0.12 *	3.6 ± 0.10	2.5 ± 0.07
Mean	3.0 ± 0.09	3.6 ± 0.09	2.8 ± 0.08	2.1± 0.06 *

Values in rows followed by the symbol “*” do differ significantly (*p* < 0.05) as assessed by the post hoc test (Tukey test). Data presented as a mean ± SD (*n* = 3).

**Table 5:** Theoretical interpretation of the IR spectra of the *C. vulgaris* and *A. platensis* lipid complexes.

No.	*C. vulgaris*	*A. platensis*	Theoretical Interpretation
	Wavenumber, cm^−1^		
1	665	-	Deformation vibration of the hydroxyl group
2	760	760	Out of plane vibrations of C–H bonds of unsaturated fragments and pendulum vibrations of CH_2_
3	870	-	Vibrations of molecules in the C=CH_2_ group
4	965	-	Stretching vibration of C–C bonds between carbons of CH_2_ groups and carbons related to unsaturated bonds
5	1065	1070	Stretching vibrations of C–C bonds of chains; C–O–P stretching vibrations
6	1095	1090	Deformation vibrations (angular) of glycerol crosslink
7	1185	1183	Stretching vibrations of C–O glycerol crosslink
8	1225	1224	Wagging vibrations of CH_2_ groups
9	1283	1258	Deformation vibrations –C–O– bonds
10	1350	-	Deformation vibrations of CH_3_ groups
11	-	1450	Scissoring vibrations of CH_2_ groups
12	1448	-	Deformation vibrations of C–H
13	1474	1450	Scissoring vibrations of CH_2_ groups
14	1523	1515	Conjugation of two or more –C=C– bonds vibrations of the hydrated carboxyl group
15	1630	1648	Stretching vibrations of C=C bonds
16	2910	2900	Stretching C–H vibrations of CH_2_ groups
17	3400	3400	Stretching vibrations of the hydroxyl group

**Table 6:** Positions of the IR absorption bands in the *C. vulgaris and A. platensis* carbohydrate complexes.

No.	*C. vulgaris*	*A. platensis*	Theoretical Interpretation
	Wavenumber, cm^−1^		
1	-	750–850	Out-of-plane deformation vibrations of C–H bonds
2	1044	1050	Stretching vibration of C–C
3	1193	1190	Stretching vibration of carbonyl C–O groups
4	-	1200	Plane deformation vibrations of C–H
5	-	1253	Stretching vibration of O–H groups
6	1400–1700	1400–1650	Stretching vibrations of C=O, C=C bands, deformation vibrations of NH (δ(N–H))
7	-	2852	Symmetrical stretching vibrations of –CH_2_– (ν_s_(CH_2_))
8	-	2925	Asymmetric stretching vibrations of –CH_2_– (ν_as_(CH_2_))
9	2915,2940	-	Stretching vibrations of –CH– (ν(CH))
10	3400	-	Stretching vibrations of the hydroxyl group
11	-	3735	Stretching vibrations of N–H (ν(NH))

## Data Availability

Not applicable.

## References

[1] MacDonnell C., Tiling K., Encomio V., van der Heide T., Teunis M., Lengkeek W., Didderen K., Bouma T.J., Inglett P.W. (2022). Evaluating a novel biodegradable lattice structure for subtropical seagrass restoration. Aquat. Bot..

[2] Mousavian Z., Safavi M., Azizmohseni F., Hadizadeh M., Mirdamadi S. (2022). Characterization, antioxidant and anticoagulant properties of exopolysaccharide from marine microalgae. AMB Expr..

[3] Song I., Kim S., Kim J., Oh H., Jang J., Jeong S.J., Baek K., Shin W.-S., Sim S.J., Jin E. (2022). Macular pigment-enriched oil production from genome-edited microalgae. Microb. Cell Fact..

[4] Dolganyuk V., Belova D., Babich O., Prosekov A., Ivanova S., Katserov D., Patyukov N., Sukhikh S. (2020). Microalgae: A Promising Source of Valuable Bioproducts. Biomolecules.

[5] Morin-Crini N., Lichtfouse E., Torri G., Crini G. (2019). Applications of chitosan in food, pharmaceuticals, medicine, cosmetics, agriculture, textiles, pulp and paper, biotechnology, and environmental chemistry. Environ. Chem. Lett..

[6] Villarruel-Lopez A., Ascencio F., Nuno K. (2017). Microalgae, a potential natural functional food source—A review. Pol. J. Food Nutr. Sci..

[7] Kadkhodeaei S., Abbasiliasi S., Shun T.J., Masoumi H.R.F., Mohamed M.S., Movahedi A., Rahim R., Ariff A.B. (2015). Enhancement of protein production by microalgae *Dunaliella salina* under mixotrophic condition using response surface methodology. RSC Adv..

[8] Goiris K., Van-Colen W., Wilches I., León-Tamariz F., De Cooman L., Muylaert K. (2015). Impact of nutrient stress on antioxidant production in three species of microalgae. Algal Res..

[9] Loganathan G., Valerie B.O., Mark L. (2018). Valuable bioproducts obtained from microalgal biomass and their commercial applications: A review. Environ. Eng. Res..

[10] Rincon S.M., Urrego N.F., Avila K.J., Romero H.M., Beyenal H. (2019). Photosynthetic activity assessment in mixotrophically cultured *Chlorella vulgaris* biofilms at various developmental stages. Algal Res..

[11] Chia S.R., Chew K.W., Zaid H.F.M., Chu D.T., Tao Y., Show P.L. (2019). Microalgal protein extraction from *Chlorella vulgaris* FSP-E using triphasic partitioning technique with sonication. Front. Bioeng. Biotechnol..

[12] Anthony J., Sivashankarasubbiah K.T., Thonthula S., Rangamaran V.R., Gopal D., Ramalingam K. (2018). An efficient method for the sequential production of lipid and carotenoids from the Chlorella Growth Factor-extracted biomass of *Chlorella vulgaris*. J. Appl. Phycol..

[13] Widowati I., Zainuri M., Kusumaningrum H.P., Susilowati R., Hardivillier Y., Leignel V., Bourgougnon N., Mouget J.-L. (2017). Antioxidant activity of three microalgae *Dunaliella salina*, *Tetraselmis chuii* and *Isochrysis galbana* clone Tahiti. IOP Conference Series: Earth and Environmental Science.

[14] Pradhan B., Nayak R., Patra S., Jit B.P., Ragusa A., Jena M. (2021). Bioactive Metabolites from Marine Algae as Potent Pharmacophores against Oxidative Stress-Associated Human Diseases: A Comprehensive Review. Molecules.

[15] Pradhan B., Patra S., Nayak R., Behera C., Dash S.R., Nayak S., Sahu B.B., Bhutia S.K., Jena M. (2020). Multifunctional role of fucoidan, sulfated polysaccharides in human health and disease: A journey under the sea in pursuit of potent therapeutic agents. Int. J. Biol. Macromol..

[16] Patra S., Praharaj P.P., Panigrahi D.P., Panda B., Bhol C.S., Mahapatra K.K., Mishra S.R., Behera B.P., Jena M., Sethi G. (2020). Bioactive compounds from marine invertebrates as potent anticancer drugs: The possible pharmacophores modulating cell death pathways. Mol. Biol. Rep..

[17] Bouzidi N., Viano Y., Ortalo-Magne A., Seridi H., Alliche Z., Daghbouche Y., Culioli G., El Hattab M. (2019). Sterols from the brown alga Cystoseira foeniculacea: Degradation of fucosterol into saringosterol epimers. Arab. J. Chem..

[18] Tanna B., Mishra A. (2018). Metabolites unravel nutraceutical potential of edible seaweeds: An emerging source of functional food. Compr. Rev. Food Sci. Food Saf..

[19] Alsenani F., Tupally K.R., Chua E.T., Eltanahy E., Alsufyani H., Parekh H.S., Schenk P.M. (2020). Evaluation of microalgae and cyanobacteria as potential sources of antimicrobial compounds. Saudi Pharm J..

[20] Bhattacharjee M. (2016). Pharmaceutically valuable bioactive compounds of algae. Asian J. Pharm. Clin. Res..

[21] Niveshika, Verma E., Mishra A.K., Singh A.K., Singh V.K. (2016). Structural Elucidation and Molecular Docking of a Novel Antibiotic Compound from Cyanobacterium *Nostoc* sp. MGL001. Front. Microbiol..

[22] Otero P., Quintana S.E., Reglero G., Fornari T., García-Risco M.R. (2018). Pressurized Liquid Extraction (PLE) as an Innovative Green Technology for the Effective Enrichment of Galician Algae Extracts with High Quality Fatty Acids and Antimicrobial and Antioxidant Properties. Mar. Drugs.

[23] Dolganyuk V., Andreeva A., Budenkova E., Sukhikh S., Babich O., Ivanova S., Prosekov A., Ulrikh E. (2020). Study of Morphological Features and Determination of the Fatty Acid Composition of the Microalgae Lipid Complex. Biomolecules.

[24] Andreeva A., Budenkova E., Babich O., Sukhikh S., Ulrikh E., Ivanova S., Prosekov A., Dolganyuk V. (2021). Production, Purification, and Study of the Amino Acid Composition of Microalgae Proteins. Molecules.

[25] Tchuenkam T., Flore T., Tiencheu B., Tenyang N., Oben A., Ashu E., Marie M., Achidi A., Marie E. (2022). Chemical and antibacterial properties of lipids extracted from some plant seeds and fruits commonly used in cosmetics. Am. J. Food Sci. Technol..

[26] Lampropoulos P.K., Gkentzi D., Tzifas S., Dimitriou G. (2021). Neonatal Sepsis Due to *Bacillus subtilis*. Cureus.

[27] Dent G. (1996). Preparation of Samples for IR Spectroscopy as KBr Disks. Int. J. Vib. Spect..

[28] Kottuparambil S., Thankamony R.L., Agusti S. (2019). Euglena as a potential natural source of value-added metabolites. A review. Algal Res..

[29] Ru I.T.K., Sung Y.Y., Jusoh M., Wahid M.E.A., Nagappan T. (2020). Chlorella vulgaris: A perspective on its potential for combining high biomass with high value bioproducts. Appl. Phycol..

[30] Garcia L.C. (2012). The promises of Chlorella vulgaris as the best alternative for biodiesel: A review. J. Nat. Stud..

[31] Yeh K.L., Chang J.S. (2012). Effects of cultivation conditions and media composition on cell growth and lipid productivity of indigenous microalga *Chlorella vulgaris* ESP-31. Bioresour. Technol..

[32] Choix F.J., de-Bashan L.E., Bashan Y. (2012). Enhanced accumulation of starch and total carbohydrates in alginateimmobilized *Chlorella* spp. induced by Azospirillum brasilense: I. Autotrophic conditions. Enzym. Microb. Technol..

[33] Abdelhedi O., Jridi M., Msaddak L., Belaid A., Nasri M., Zouari N., Fakhfakh N. (2019). Spirulina (*Arthrospira*) Platensis as a Techno-Functional Ingredient in Almond Paste Process Production. Austin. J. Nutri. Food Sci..

[34] Zielinski D., Fraczyk J., Debowski M., Zielinski M., Kaminski Z., Kregiel D., Jacob C., Kolesinska B. (2020). Biological Activity of Hydrophilic Extract of *Chlorella vulgaris* Grown on Post-Fermentation Leachate from a Biogas Plant Supplied with Stillage and Maize Silage. Molecules.

[35] Frazzini S., Scaglia E., Dell’Anno M., Reggi S., Panseri S., Giromini C., Lanzoni D., Sgoifo Rossi C.A., Rossi L. (2022). Antioxidant and Antimicrobial Activity of Algal and Cyanobacterial Extracts: An In Vitro Study. Antioxidants.

[36] Selivanova E.A., Ignatenko M.E., Nemtseva N.V. (2014). Antagonistic activity of novel green microalgae strain. Zh. Mikrobiol. Epidemiol. Immunobiol..

[37] Pina-Pérez M.C., Rivas A., Martínez A., Rodrigo D. (2017). Antimicrobial potential of macro and microalgae against pathogenic and spoilage microorganisms in food. Food Chem..

[38] Singh M., Singh S., Prasad S., Gambhir I. (2008). Nanotechnology in medicine and antibacterial effect of silver nanoparticles. Digest J. Nanomater. Biostructures.

[39] Mostafa S.M.S., Kumar D.N. (2012). Microalgal biotechnology: Prospects and applications. Plant Science.

[40] Ahmad M.T., Shariff M., Yusoff F.M., Goh Y.M., Banerjee S. (2018). Applications of microalga *Chlorella vulgaris* in aquaculture. Rev. Aquac..

[41] Safi C., Zebib B., Merah O., Pontalier P. (2014). Morphology, composition, production, processing and applications of *Chlorella vulgaris*: A review. Renew. Sustain. Energy Rev..

[42] Tabarsa M., Shin I.S., Lee J.H., Surayot U., Park W., You S. (2015). An immune-enhancing water-soluble α glucan from *Chlorella vulgaris* and structural characteristics. Food Sci. Biotechnol..

[43] Scott A.M., Beller E., Glasziou P., Clark J., Ranakusuma R.W., Byambasuren O., Bakhit M., Page S.W., Trott D., Del Mar C. (2018). Is Antimicrobial Administration to Food Animals a Direct Threat to Human Health? A Rapid Systematic Review. Int. J. Antimicrob. Agents.

[44] Sharma C., Rokana N., Chandra M., Singh B.P., Gulhane R.D., Gill J.P.S., Ray P., Puniya A.K., Panwar H. (2018). Antimicrobial Resistance: Its Surveillance, Impact, and Alternative Management Strategies in Dairy Animals. Front. Vet. Sci..

[45] Caprarulo V., Hejna M., Giromini C., Liu Y., Dell’Anno M., Sotira S., Reggi S., Sgoifo-Rossi C.A., Callegari M.L., Rossi L. (2020). Evaluation of Dietary Administration of Chestnut and Quebracho Tannins on Growth, Serum Metabolites and Fecal Parameters of Weaned Piglets. Animals.

[46] Ricky R., Chiampo F., Shanthakumar S. (2022). Efficacy of Ciprofloxacin and Amoxicillin Removal and the Effect on the Biochemical Composition of *Chlorella vulgaris*. Bioengineering.

[47] Bhuvaneswari G.R., Shukla S.P., Makesh M., Thirumalaiselvan S., Sudhagar S.A., Kothari D.C., Singh A. (2013). Antibacterial Activity of Spirulina (*Arthospira platensisGeitler*) against Bacterial Pathogens in Aquaculture. Isr. J. Aquac.-Bamidgeh.

[48] Daglia M. (2012). Polyphenols as antimicrobial agents. Curr. Opin. Biotechnol..

[49] Etahiri S., Bultel-Poncé V., Caux C., Guyot M. (2001). New Bromoditerpenes from the Red Alga Sphaerococcus Coronopifolius. J. Nat. Prod..

[50] Darias J., Rovirosa J., San Martin A., Díaz A.-R., Dorta E., Cueto M. (2001). Furoplocamioids A−C, Novel Polyhalogenated Furanoid Monoterpenes from Plocamium c Artilagineum. J. Nat. Prod..

[51] Barreto M., Meyer J.J.M. (2006). Isolation and Antimicrobial Activity of a Lanosol Derivative from Osmundaria Serrata (Rhodo-phyta) and a Visual Exploration of Its Biofilm Covering. S. Afr. J. Bot..

[52] Kavita K., Singh V.K., Jha B. (2014). 24-Branched ∆5 Sterols from Laurencia Papillosa Red Seaweed with Antibacterial Activity against Human Pathogenic Bacteria. Microbiol. Res..

[53] das Neves dos Santos Amorim R., Rodrigues J.A.G., Holanda M.L., Quinderé A.L.G., de Paula R.C.M., Melo V.M.M., Benevides N.M.B. (2012). Antimicrobial Effect of a Crude Sulfated Polysaccharide from the Red Seaweed Gracilaria Ornata. Braz. Arch. Biol. Technol..

[54] Stabili L., Acquaviva M.I., Biandolino F., Cavallo R.A., de Pascali S.A., Fanizzi F.P., Narracci M., Petrocelli A., Cecere E. (2012). The Lipidic Extract of the Seaweed Gracilariopsis Longissima (Rhodophyta, Gracilariales): A Potential Resource for Bio-technological Purposes?. New Biotechnol..

[55] Abdel-Moneim A.-M.E., El-Saadony M.T., Shehata A.M., Saad A.M., Aldhumri S.A., Ouda S.M., Mesalam N.M. (2022). Antioxi-dant and Antimicrobial Activities of Spirulina Platensis Extracts and Biogenic Selenium Nanoparticles against Selected Pathogenic Bacteria and Fungi. Saudi J. Biol. Sci..

[56] Zhao T., Han X., Cao H. (2020). Effect of Temperature on Biological Macromolecules of Three Microalgae and Application of FT-IR for Evaluating Microalgal Lipid Characterization. ACS Omega.

[57] Nosenko T., Sitnikova V., Strelnikova I., Fokina M. (2021). Workshop on Vibrational Spectroscopy.

[58] Alghazeer R., Whida F., Abduelrhman E., Gammoudi F., Azwai S. (2013). Screening of antibacterial activity in marine green, red and brown macroalgae from the western coast of Libya. Nat. Sci..

[59] Sun H., Song W., Zhang L., Yang X., Zhu Z., Ma R., Wang D. (2018). Structural characterization and inhibition on Į-glucosidase of a novel oligosaccha-ride from barley malt. J. Cereal. Sci..

[60] Wang C., Zhang D., Zhang M., Jiao Y., Jiang K., Yan C. (2017). Structural characterization of a novel oligosaccharide from Achyranthes bi-dentata and its anti-osteoporosis activities. Ind. Crop Prod.

[61] Meng Y., Yao C., Xue S., Yang H. (2014). Application of Fourier transform infrared (FT-IR) spectroscopy in determination of mi-croalgal compositions. Bioresour. Technol..

[62] Dalal S.R., Hussein M.H., El-Naggar N.E.-A., Mostafa S.I., Shaaban-Dessuuki S.A. (2021). Characterization of alginate extracted from *Sargassum latifolium* and its use in *Chlorella vulgaris* growth promotion and riboflavin drug delivery. Sci. Rep..

[63] Flórez-Fernández N., Domínguez H., Torres M.D. (2019). A green approach for alginate extraction from Sargassum muticum brown seaweed using ultrasound-assisted technique. Int. J. Biol. Macromol..

[64] Aprilliza M. (2017). Characterization and properties of sodium alginate from brown algae used as an ecofriendly superabsorbent. IOP Conference Series: Materials Science and Engineering.

[65] Vidyadharani G., Dhandapani R. (2013). Fourier transform infrared (FTIR) spectroscopy for the analysis of lipid from *Chlorella vulgaris*. Elixir Appl. Biol..

[66] Stojanovic R., Belscak-Cvitanovic A., Manojlovic V., Komes D., Nedovic V., Bugarski B. (2012). Encapsulation of thyme (*Thymus serpyllum* L.) aqueous extract in calcium alginate beads. J. Sci. Food Agric..

[67] Carpenter J., Saharan V.K. (2017). Ultrasonic assisted formation and stability of mustard oil in water nanoemulsion: Effect of pro-cess parameters and their optimization. Ultrason. Sonochem..

[68] Cardenas-Jiron G., Leal D., Matsuhiro B., Osorio-Roman I.O. (2011). Vibrational spectroscopy and density functional theory cal-culations of poly-D-mannuronate and heteropolymeric fractions from sodium alginate. J. Raman Spectrosc..

[69] Bouissil S., El Alaoui-Talibi Z., Pierre G., Michaud P., El Modafar C., Delattre C. (2020). Use of alginate extracted from Moroccan brown algae to stimulate natural defense in date palm roots. Molecules.

[70] Gomaa M., Fawzy M.A., Hifney A.F., Abdel-Gawad K.M. (2018). Use of the brown seaweed *Sargassum latifolium* in the design of alginate-fucoidan based films with natural antioxidant properties and kinetic modeling of moisture sorption and polyphenolic release. Food Hydrocoll..

[71] El-Naggar N.E.-A., Hussein M.H., Shaaban-Dessuuki S.A., Dalal S.R. (2020). Production, ex-traction and characterization of *Chlorella vulgaris* soluble polysaccharides and their ap-plications in AgNPs biosynthesis and biostimulation of plant growth. Sci. Rep..

[72] Activityradhika D., Mohaideen A. (2015). Transform infrared analysis of ulva lactuca and gracilaria corticata and their effect on antibacterial. Asian J. Pharm. Clin. Res..

[73] AftabUddin S., Siddique M.A.M., Habib A., Akter S., Hossen S., Tan-changya P., Al M.A. (2021). Effects of seaweeds extract on growth, survival, antibacterial activities, and immune re-sponses of *Penaeus monodon* against *Vibrio parahaemolyticus*. Ital. J. Anim. Sci..

[74] Samper-Villarreal J., Moya-Ramírez J., Cortés J. (2022). First characterization of seagrasses at Sámara Bay, Pacific coast of Costa Rica. Aquat. Bot..

[75] Thibaut T., Blanfuné A., Boudouresque C.F., Holon F., Agel N., Descamps P., Deter J., Pavy T., Delaruelle G., Verlaque M. (2022). Distribution of the seagrass Halophila stipulacea: A big jump to the northwestern Mediterranean Sea. Aquat. Bot..

